# Spatiotemporal Analysis of HIV/AIDS Incidence in China From 2009 to 2019 and Its Association With Socioeconomic Factors: Geospatial Study

**DOI:** 10.2196/56229

**Published:** 2024-06-07

**Authors:** Ziyi Xie, Bowen Chen, Zhizhuang Duan

**Affiliations:** 1 Faculty of Humanities and Social Science Macao Polytechnic University Macao China; 2 Xingzhi College Zhejiang Normal University Jinhua China

**Keywords:** HIV/AIDS, spatiotemporal distribution, cluster analysis, socioeconomic factors, China

## Abstract

**Background:**

The Joint United Nations Program on HIV/AIDS (UNAIDS) has set the “95-95-95” targets to ensure that 95% of all people living with HIV will know their HIV status, 95% of all people living with HIV will receive sustained antiretroviral therapy (ART), and 95% of all people receiving ART will achieve viral suppression (<1000 copies/mL). However, few countries have currently achieved these targets, posing challenges to the realization of the UNAIDS goal to eliminate the global HIV/AIDS epidemic by 2030. The Chinese government has implemented corresponding policies for HIV/AIDS prevention and control; however, it still faces the challenge of a large number of HIV/AIDS cases. Existing research predominantly focuses on the study of a particular region or population in China, and there is relatively limited research on the macro-level analysis of the spatiotemporal distribution of HIV/AIDS across China and its association with socioeconomic factors.

**Objective:**

This study seeks to identify the impact of these factors on the spatiotemporal distribution of HIV/AIDS incidence in China, aiming to provide scientific recommendations for future policy development.

**Methods:**

This study employed ArcGIS 10.2 (Esri) for spatial analysis, encompassing measures such as the imbalance index, geographical concentration index, spatial autocorrelation analysis (Moran I), and hot spot analysis (Getis-Ord Gi*). These methods were used to unveil the spatiotemporal distribution characteristics of HIV/AIDS incidence in 31 provinces of China from 2009 to 2019. Geographical Detector was used for ecological detection, risk area detection, factor detection, and interaction detection. The analysis focused on 9 selected socioeconomic indicators to further investigate the influence of socioeconomic factors on HIV/AIDS incidence in China.

**Results:**

The spatiotemporal distribution analysis of HIV/AIDS incidence in China from 2009 to 2019 revealed distinct patterns. The spatial distribution type of HIV/AIDS incidence in China was random in 2009-2010. However, from 2011 to 2019, the distribution pattern evolved toward a clustered arrangement, with the degree of clustering increasing each year. Notably, from 2012 onwards, there was a significant and rapid growth in the aggregation of cold and hot spot clusters of HIV/AIDS incidence in China, stabilizing only by the year 2016. An analysis of the impact of socioeconomic factors on HIV/AIDS incidence in China highlighted the “urbanization rate” and “urban basic medical insurance fund expenditure” as the primary factors influencing the spatial distribution of HIV/AIDS incidence. Additionally, among social factors, indicators related to medical resources exerted a crucial influence on HIV/AIDS incidence.

**Conclusions:**

From 2009 to 2019, HIV/AIDS incidence in China was influenced by various socioeconomic factors. In the future, it is imperative to optimize the combination of different socioeconomic indicators based on regional incidence patterns. This optimization will facilitate the formulation of corresponding policies to address the challenges posed by the HIV/AIDS epidemic.

## Introduction

The Joint United Nations Program on HIV/AIDS (UNAIDS) has set the “95-95-95” targets to be achieved by 2025 [1], aiming for 95% of all people living with HIV/AIDS to be aware of their HIV status, 95% of people living with HIV/AIDS to receive sustained antiretroviral therapy (ART), and 95% of those receiving ART to achieve viral suppression (<1000 copies/mL) [2]. The global goal is to eliminate the HIV/AIDS epidemic worldwide by 2030, which is a public health threat [3]. However, a recent report by the UNAIDS titled “The path that ends AIDS: UNAIDS Global AIDS Update 2023” revealed that only 5 countries (Botswana, Eswatini, Rwanda, the United Republic of Tanzania, and Zimbabwe) have achieved the “95-95-95” targets ahead of schedule [4]. China, which is a middle-income country with a relatively high number of people living with HIV/AIDS [3], plays a crucial role in advancing the comprehensive realization of the “95-95-95” targets. Since the report of the first AIDS-related death in China in 1985 [5], the Chinese government has implemented a series of policies for the prevention and control of the HIV/AIDS epidemic, achieving certain successes [6]. However, due to its vast geographical expanse and large population, the HIV/AIDS epidemic in China, despite having an overall low prevalence rate [7], still presents a significant challenge for public health governance due to the substantial number of reported cases [8-10]. An epidemiological analysis of HIV/AIDS in China from 2004 to 2016 also confirmed the escalating severity of the epidemic and the significant regional disparities in HIV/AIDS infection rates [11]. Therefore, for HIV/AIDS prevention and control in China, it is particularly crucial to accurately identify high-risk areas within its vast territory and tailor HIV/AIDS prevention and control policies accordingly. The widespread application of geographic information technology in the field of epidemiological surveillance offers new avenues for addressing this challenge [12,13].

Existing studies have indicated a close correlation between the prevention and control of HIV/AIDS and both geographical spatial factors [14] and socioeconomic elements [15]. For instance, a study using gridded demographic data sets for spatial microsimulation to map HIV/AIDS indicators in low- and middle-income countries emphasized the need for appropriate socioeconomic categorization of data to explain social spatial disparities [16]. Another study conducted geographical clustering analysis of social capital distribution data and HIV/AIDS incidence, revealing that the spatial distribution of social capital would significantly impact HIV/AIDS care and prevention efforts [17]. Additionally, an analysis of the geographical distribution disparities in HIV mortality rates in South Africa identified more pronounced local spatial clustering characteristics in periurban areas and around national highways, and this finding was attributed to these areas being population clusters but lacking sufficient economic support for people living with HIV/AIDS to seek medical assistance [18]. Furthermore, 2 studies on the clustering analysis of HIV mortality rates among children and pregnant women further confirmed this viewpoint [19,20]. From this, it can be seen that studies in other countries have already linked these factors to the prevention and control of HIV/AIDS. However, there is currently a scarcity of research in academia that analyzes the HIV epidemic in China based on these 2 categories of factors, and the limited research conducted often focuses on the spatiotemporal analysis of data from specific regions or populations. For instance, in terms of geographic selection, the majority of current spatiotemporal analyses of HIV/AIDS in China targeted specific regions, with little detailed discussion on the correlation between spatiotemporal factors and socioeconomic factors [21-23]. While some studies have conducted spatiotemporal analyses of HIV/AIDS data across China, they have not linked the findings to socioeconomic factors [24,25]. Additionally, in some spatiotemporal analyses based on the entire HIV/AIDS population in China, characteristics of specific groups, such as youth and men who have sex with men, have been studied. Although these findings have been connected to macroscopic socioeconomic factors, the degree of the influence of corresponding microscopic indicators under these factors on spatiotemporal distribution has not been thoroughly explored [26-28]. This provides research space for our study to further investigate the correlation between the spatiotemporal distribution characteristics of HIV/AIDS incidence across China and the subdivision indicators under socioeconomic factors.

Additionally, it is worth emphasizing that, while the COVID-19 pandemic in China in 2020 posed a threat to HIV/AIDS treatment and health care services [29], the stringent lockdown measures may have influenced the incidence of HIV/AIDS differently [30]. Research in the context of this sudden public health event needs to be carried out separately to ensure scientific rigor. Therefore, this study specifically selected data on the incidence of HIV/AIDS in China before the occurrence of the COVID-19 pandemic. It is important to note that the standard system for assessing the HIV/AIDS epidemic includes infection and mortality data. The reason for choosing the incidence of HIV/AIDS as the core criterion in this study instead of the infection rate is that the infection rate involves a considerably long incubation period for HIV and issues related to voluntary or mandatory testing [31]. Meanwhile, the mortality rate is, in a sense, an extension of the incidence rate. In terms of selecting socioeconomic indicators, a study on the spatiotemporal distribution and influencing factors of HIV/AIDS incidence in China found that the level of economic development (gross domestic product [GDP]) is the most closely related factor [32]. Another study further demonstrated that, in addition to overall economic conditions, factors, such as the urbanization rate and population density, are closely correlated with HIV/AIDS incidence [33]. Furthermore, several other studies on the access of people living with HIV/AIDS to health care services have shown that the accessibility of medical services [34] and individual-level economic conditions [35,36] also impact HIV/AIDS incidence, and this situation can be improved if the government continues to increase public health expenditure [37]. Building upon the aforementioned existing research, we combined statistical data published by the China National Bureau of Statistics (CNBS) with the actual situation of HIV/AIDS prevention and control in China, ultimately selecting 9 representative socioeconomic indicators as variables.

Taking into consideration the aforementioned factors, this study initially employed the imbalance index, geographical concentration index, spatial autocorrelation analysis (Moran I), and hot spot analysis (Getis-Ord Gi*) in ArcGIS 10.2 (Esri). These analyses aim to reveal the spatiotemporal distribution characteristics of HIV/AIDS incidence (both the number of cases and incidence rate per 100,000 people) in 31 provinces of China from 2009 to 2019. Subsequently, Geographical Detector was employed to conduct ecological detection, risk area detection, factor detection, and interaction detection. This analysis explored the impact of 9 indicators related to China’s social and economic factors on the spatiotemporal distribution of HIV/AIDS incidence. The aim was to provide policy recommendations for China to further address the HIV/AIDS epidemic effectively.

## Methods

### Data Collection

This study integrated HIV/AIDS incidence data from 2009 to 2019 in 31 provinces of China (excluding Hong Kong, Macao, and Taiwan) with socioeconomic indicators to facilitate a comprehensive analysis and causal discussion in both temporal and spatial dimensions. Specifically, social indicators included the number of health care institutions, number of beds in health care institutions, number of health care personnel, urbanization rate, and residential population density. Economic indicators included GDP, expenditure on health care in local finances, per capita disposable income of residents, and urban basic medical insurance fund expenditure. The socioeconomic data were sourced from the CNBS, while the HIV/AIDS incidence data were obtained from the Chinese Public Health Science Data Center (CPHSD). Specifically, the HIV/AIDS incidence data from CPHSD are compiled based on the Chinese Infectious Disease Reporting System. Local hospitals and Centers for Disease Control and Prevention (CDC) regularly report newly diagnosed HIV/AIDS cases through this network system and calculate HIV/AIDS incidence, which is then used by the Chinese Center for Disease Control and Prevention (CCDC) to compile national HIV/AIDS data [38]. Spatial administrative boundary vector data are derived from the China basic geographic information data released by the National Geomatics Center of China (NGCC).

Furthermore, to eliminate differences in dimensional units among various indicators and meet the requirements of the Geographical Detector for operational data, the Natural Breaks method in ArcGIS 10.2 software was employed to categorize the independent variables. Each socioeconomic indicator was divided into 5 levels, represented by X_1_ to X_9_ (Table 1).

**Table 1:** Socioeconomic indicators (factors) influencing the incidence of HIV/AIDS in China.

Influencing indicator	Code	Grade
Gross domestic product	X_1_	5
Expenditure on health care in local finances	X_2_	5
Per capita disposable income of residents	X_3_	5
Urban basic medical insurance fund expenditure	X_4_	5
Number of health care institutions	X_5_	5
Number of beds in health care institutions	X_6_	5
Number of health care personnel	X_7_	5
Urbanization rate	X_8_	5
Residential population density	X_9_	5

### Ethical Considerations

The study used original data sourced from the CPHSD, an official government institution, which publicly releases data. According to the “Ethical Review Measures for Human Life Science and Medical Research” issued by the National Health Commission of China and the “Scientific Data Management Measures” issued by the General Office of the State Council of the People’s Republic of China, researchers are permitted to conduct secondary analysis of publicly available anonymized data for scientific research purposes without requiring Institutional Review Board (IRB) scrutiny and approval. These regulations exempt research involving pre-existing data that are publicly available or that are recorded in a manner that subjects cannot be identified directly or through identifiers linked to the subjects. Given that the data used in our study were both anonymized and publicly available, IRB review was not necessary for the study. Additionally, the authors have obtained permission to access and use the data, ensuring adherence to data usage policies and ethical standards.

### Statistical Methods

#### Imbalance Index

The imbalance index is a crucial indicator that illustrates the degree of distribution balance of the research subjects in different regions [39]. The imbalance index (S) takes values between 0 and 1, with a larger S value indicating a greater imbalance in the distribution of HIV/AIDS incidence within the study area. Its calculation formula is as follows:

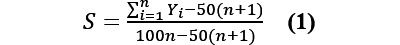



#### Geographical Concentration Index

The geographical concentration index is a crucial indicator that indicates the degree of geographical concentration of the research object [40]. The geographical concentration index (G) takes values between 0 and 100. A higher G value indicates a more concentrated spatial distribution of HIV/AIDS incidence in China, while a lower value suggests a more dispersed spatial distribution. Its calculation formula is as follows:

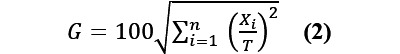



#### Spatial Autocorrelation Analysis (Moran I)

The Moran I index is a significant indicator for analyzing the spatial distribution relationships among units in the study area [41]. It can be used to identify and measure the distribution status and clustering degree of HIV/AIDS incidence in the 31 provinces of China. An I value >0 indicates positive spatial autocorrelation among the units, while a value <0 indicates negative spatial autocorrelation. A larger I value signifies a more significant spatial correlation. The significance of the Moran I index is generally tested using the Z-score formula. When the Z-score exceeds the critical value of 1.96, it indicates the statistical significance of spatial clustering, with the probability of randomly generating this clustering pattern being less than 5%. The calculation formula is as follows:

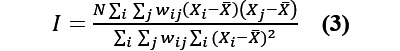



#### Hot Spot Analysis (Getis-Ord Gi*)

Hot spot analysis is an important analytical method that explores the similarity and heterogeneity between the attribute values of spatial geographic units and their adjacent units [42]. This method compares the local sum of a particular feature and its adjacent features with the total sum of all features. When the local sum of an area significantly differs from the expected local sum to the extent that it cannot be attributed to random chance, the area is identified as a high- or low-value cluster (hot or cold spot) based on the attribute values. This facilitates the visualization of the spatial distribution of HIV/AIDS incidence. The calculation formula is as follows:

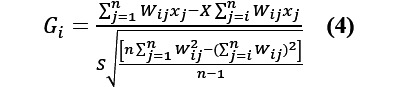



#### Geographical Detector

The Geographical Detector encompasses 4 detection modes (ecological detection, risk area detection, factor detection, and interaction detection) used to uncover the driving factors behind the spatial distribution of a particular phenomenon [43]. It is applied for detecting and assessing HIV/AIDS incidence. Ecological detection, in this context, aims to determine if there is a significant difference in the spatial distribution impact on attribute Y between 2 factors, X_1_ and X_2_. The calculation formula is as follows:

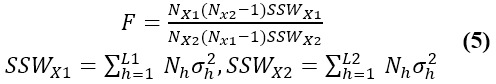



Among these, risk area detection is used to assess whether there is a significant difference in the mean HIV/AIDS incidence between 2 subregions. The calculation formula is as follows:

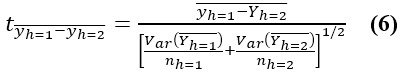



Factor detection is a method used to assess the extent to which the 9 socioeconomic indicators can explain the spatial variation of HIV/AIDS incidence. The calculation formula is as follows:

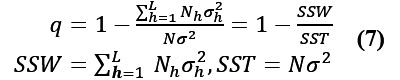



Interaction detection is primarily employed to identify the interactions among the 9 socioeconomic indicators (Xs). It assesses whether the joint effect of any 2 socioeconomic indicators increases or decreases the explanatory power regarding HIV/AIDS incidence. Additionally, it examines whether the impact of these indicators on HIV/AIDS incidence is mutually independent. The results of the interaction can be categorized into the following 5 types: nonlinear attenuation, si-factor nonlinear attenuation, bi-factor authentication, mutually independent, and nonlinear enhancement (Table 2).

**Table 2:** Types of interactions among socioeconomic indicators (factors) influencing the incidence of HIV/AIDS in China.

Basis for judgment	Type of interaction
q(X_1_ ∩ X_2_) < Min(q(X_1_),q(X_2_))	Nonlinear attenuation
Min(q(X_1_),q(X_2_)) < q(X_1_ ∩ X_2_) < Max(q(X_1_),q(X_2_))	Si-factor nonlinear attenuation
q(X_1_ ∩ X_2_) > Max(q(X_1_),q(X_2_))	Bi-factor authentication
q(X_1_ ∩ X_2_) = q(X_1_) + q(X_2_)	Mutually independent
q(X_1_ ∩ X_2_) > q(X_1_) + q(X_2_)	Nonlinear enhancement

## Results

### Spatiotemporal Distribution Characteristics of HIV/AIDS Incidence in China

This study initially used the imbalance index and geographical concentration index to measure the equilibrium and concentration of the spatiotemporal distribution characteristics of HIV/AIDS incidence in 31 provinces of China from 2009 to 2019. Subsequently, spatial autocorrelation analysis (Moran I ) was introduced to account for the spatial proximity of the study objects, further specifying the spatial distribution types of HIV/AIDS incidence from 2009 to 2019. Finally, hot spot analysis was employed to visually depict the evolutionary characteristics of the spatiotemporal distribution of HIV/AIDS incidence in China from 2009 to 2019.

Based on the calculation results of the imbalance index and geographical concentration index (Table 3), there was a slight downward trend in both indices from 2013 to 2018, but the values remained stable within a certain range. Overall, the imbalance index (S) stabilized in the range of 0.4-0.5, and the geographical concentration index (G) stabilized in the range of 24-34, indicating that the spatiotemporal distribution of HIV/AIDS incidence in China from 2009 to 2019 exhibited a certain degree of imbalance and concentration. Moreover, this distribution state demonstrated a certain level of persistence and stability over time.

**Table 3:** Imbalance index and geographical concentration index of HIV/AIDS incidence in China from 2009 to 2019.

Year	HIV incidence rate (/100,000 people)	Imbalance index (S)	Geographical concentration index (G)
2009	1.00	0.58	34.68
2010	1.20	0.54	32.09
2011	1.53	0.53	31.77
2012	3.11	0.54	30.25
2013	3.12	0.49	27.53
2014	3.33	0.46	26.28
2015	3.69	0.42	24.72
2016	3.97	0.40	24.13
2017	4.14	0.41	24.30
2018	4.62	0.41	24.81
2019	5.10	0.44	25.89

Using the spatial autocorrelation analysis tool in ArcGIS 10.2, the Moran I value for the spatial distribution of HIV/AIDS incidence in China from 2009 to 2019 was obtained (Table 4). The results indicated that the Moran I value was only 0.05 in 2009-2010, suggesting a random spatial distribution pattern for HIV/AIDS incidence in China during that period. In 2011, the Moran I value increased to 0.07 and passed the significance test at the 5% level, indicating a transition to a clustered distribution pattern. Subsequently, the Moran I value continued to rise, with an increase from 0.07 to 0.25, demonstrating strong positive spatial autocorrelation, and it consistently passed the significance test at the 5% level. This confirms that the distribution pattern of HIV/AIDS incidence in China evolved from a random distribution to a clustered distribution from 2011 to 2019, with the degree of clustering increasing annually, demonstrating strong spatial aggregation and dependence.

**Table 4:** Moran I values and spatial distribution types of HIV/AIDS incidence in China from 2009 to 2019.

Year	Moran I	Z-score	*P* value	Distribution type^a^
2009	0.05	1.44	.14	Random
2010	0.05	1.43	.15	Random
2011	0.07	1.71	.09	Clustered
2012	0.11	2.05	.04	Clustered
2013	0.14	2.48	.01	Clustered
2014	0.16	2.67	.01	Clustered
2015	0.19	2.97	<.001	Clustered
2016	0.22	3.32	<.001	Clustered
2017	0.23	3.51	<.001	Clustered
2018	0.23	3.55	<.001	Clustered
2019	0.25	3.77	<.001	Clustered

^a^Evolution from a random distribution to a clustered distribution is indicated.

**Figure 1:**
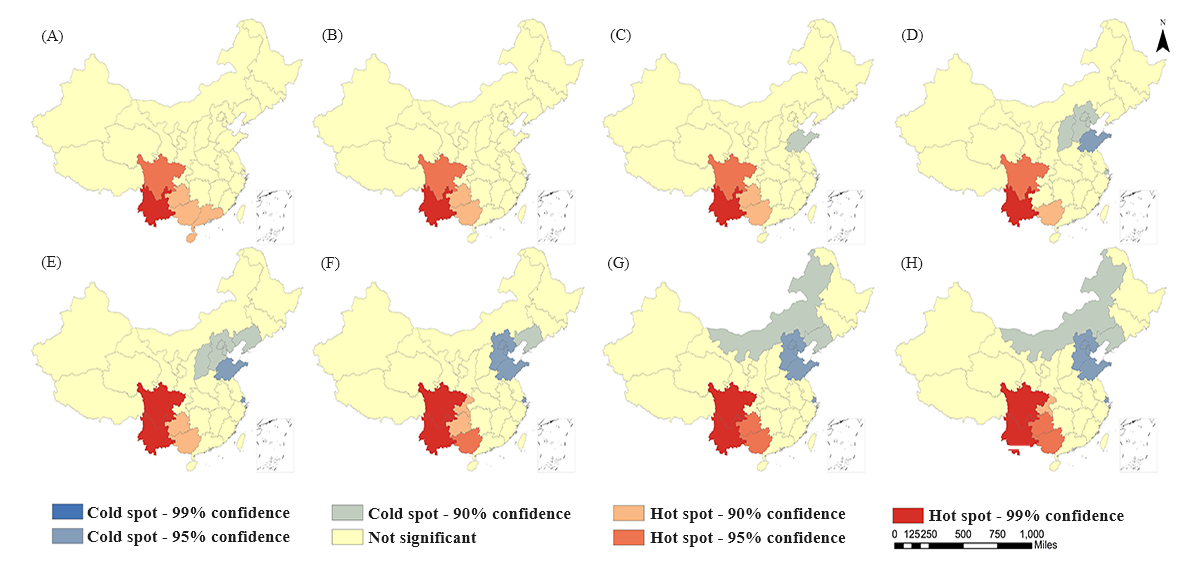
Spatial distribution map of hot and cold spots of HIV/AIDS incidence in China from 2009 to 2019. (A) 2009, (B) 2010, (C) 2011, (D) 2012, (E) 2013, (F) 2014, (G) 2015, (H) 2016-2019.

For the study of spatial distribution characteristics, areas with higher or lower attribute values are often the first to attract attention. However, the high or low values of attributes do not necessarily represent statistically significant hot or cold spots [40]. Therefore, areas that can be considered hot or cold spots should have features with high values (low values) and be surrounded by other features that also have high values (low values). To more precisely illustrate these spatial distribution relationships, this study used the Hot Spot Analysis tool in ArcGIS 10.2 to conduct Getis-Ord Gi* statistical analysis. The analysis identified hot and cold spots in the spatial clustering distribution of HIV incidence in China from 2009 to 2019 (Figure 1). It should be noted that during the data analysis process, we found that the distribution of hot and cold spots did not change from 2016 to 2019. Therefore, the distribution status of hot and cold spots from 2016 to 2019 has been combined and presented as 1 map in Figure 1H.

The number of cold spot clusters of HIV incidence in China started from 0 in 2009-2010 and experienced significant and rapid growth in 2012. Although there was a slight decline in the number of cold spots in 2014, it did not prevent the overall stability of cold spot clusters at 6-7 from 2012 to 2019. In general, from 2009 to 2019, the spatial extent of low-value cold spot areas expanded significantly, spreading from Shandong Province to the central and northern provinces of China. Ultimately, these cold spot clusters stabilized and concentrated in the central and northern provinces of China (Shandong, Beijing, Tianjin, Hebei, Shanxi, Liaoning, and Inner Mongolia).

In terms of hot spot clusters, the number of hot spot clusters of HIV/AIDS incidence in China showed a trend of first decreasing and then increasing, but the magnitude of the increase and decrease was not significant. It mainly stabilized in 5 southwestern provinces of China (Yunnan, Guangxi, Sichuan, Guizhou, and Chongqing), with an improvement in both aggregation and significance. Overall, compared to cold spot areas, hot spot areas exhibited a smaller fluctuation range and stronger spatial distribution stability.

### Impact of Socioeconomic Factors on the Spatiotemporal Distribution of HIV/AIDS Incidence in China

HIV/AIDS, as an infectious disease, is profoundly influenced by socioeconomic factors in its spatiotemporal distribution [44]. This study selected 9 socioeconomic indicators related to HIV/AIDS incidence and employed the Geographical Detector for quantitative exploration, aiming to analyze the extent of the impact of these indicators on the spatial distribution of HIV/AIDS incidence in China.

The results of risk detection and ecological detection indicated, at a significance level of 0.05, that there were no significant differences in the mean values of attributes between subregions of different levels. Among the socioeconomic indicators, only the urbanization rate, per capita disposable income, and population density exhibited significantly different impact capacities. There was no significant difference in the impact of other indicators on the spatial distribution of HIV/AIDS incidence in China. This suggests a certain rationality in the selection and grading of indicators.

The results of factor detection showed the strength of the impact of various factors on the spatial distribution of HIV/AIDS incidence, with the magnitude of q values as follows (Table 5): urbanization rate (X_8_=0.49) > urban basic medical insurance fund expenditure (X_4_=0.31) > the number of beds in health care institutions (X_6_=0.28) > local financial expenditure on health care (X_2_=0.26) > the number of health care personnel (X_7_=0.26) > the number of health care institutions (X_5_=0.19) > provincial GDP (X_1_=0.12) > nonsignificant impacts for per capita disposable income and population density (X_3_=0.05 and X_9_=0.06, respectively).

**Table 5:** Factor detection results of socioeconomic indicators (factors) regarding HIV/AIDS incidence in China from 2009 to 2019.

Indicator^a^	q statistic	*P* value
X_1_	0.12	.60
X_2_	0.26	.19
X_3_	0.05	.85
X_4_	0.31	.17
X_5_	0.19	.35
X_6_	0.28	.15
X_7_	0.26	.19
X_8_	0.49	.01
X_9_	0.06	.80

^a^The indicators are clarified in Table 1.

Based on the above detection results, it can be concluded that the urbanization rate and the expenditure on urban basic medical insurance were the most influential factors explaining the spatial distribution of HIV/AIDS incidence in China. Provinces with low urbanization rates and minimal spending on urban basic medical insurance were more likely to face a severe situation of HIV/AIDS incidence. The number of beds in health care institutions, local financial expenditure on health care, number of health care personnel, and number of health care institutions were secondary factors influencing the distribution. These 4 indicators belong to health care resource factors within social indicators, indicating that a better health care development situation has a certain inhibitory effect on the distribution of HIV/AIDS incidence in China. GDP had a certain explanatory power for the distribution of the incidence but with a low *P* value, suggesting lower confidence in its explanatory power and indicating a correlation (not significant) with the spatial distribution of HIV/AIDS incidence in China. Additionally, per capita disposable income and population density had a relatively weak impact on the spatial distribution pattern of HIV/AIDS incidence in China.

The results of interactive detection are shown in Table 6. After the interaction of any 2 of the 9 socioeconomic indicators, all q-values exhibited varying degrees of increase, showing nonlinear enhancement or bivariate enhancement states, with no independent or weakening relationships. This suggests that, compared to single indicators, the coupling effect of any 2 factors can enhance the explanatory power of the spatiotemporal distribution pattern of HIV/AIDS incidence in China, indicating that the spatial distribution differences of HIV/AIDS incidence in China are the result of the interaction of multiple influencing factors. According to the analysis results of ecological detection, the differences in the effects of each indicator were not statistically significant, which indicates the significant importance of the comprehensive synergistic effect of multiple factors in exploring HIV/AIDS incidence.

**Table 6:** Interaction detection results of socioeconomic indicators (factors) regarding HIV/AIDS incidence in China from 2009 to 2019.

Indicator^a,b^	X_1_	X_2_	X_3_	X_4_	X_5_	X_6_	X_7_	X_8_	X_9_
X_1_	0.12	0.67	0.36	0.51	0.34	0.47	0.60	0.81	0.23
X_2_	0.67	0.26	0.67	0.67	0.45	0.38	0.32	0.75	0.73
X_3_	0.36	0.67	0.05	0.62	0.38	0.55	0.55	0.59	0.17
X_4_	0.51	0.67	0.62	0.31	0.55	0.66	0.55	0.80	0.71
X_5_	0.34	0.45	0.38	0.55	0.19	0.40	0.35	0.72	0.31
X_6_	0.47	0.38	0.55	0.66	0.40	0.28	0.33	0.75	0.57
X_7_	0.60	0.32	0.55	0.55	0.35	0.33	0.26	0.75	0.68
X_8_	0.81	0.75	0.59	0.80	0.72	0.75	0.75	0.49	0.67
X_9_	0.23	0.73	0.17	0.71	0.31	0.57	0.68	0.67	0.06

^a^The indicators are clarified in Table 1.

^b^The diagonal values in the table represent the q-values of single factors, while the other values denote interactions between factors.

Furthermore, among all combinations, the enhancement effect of the urbanization rate with other indicators was the most significant. The output results of ecological detection also indicated that only the urbanization rate exhibited a significant difference with other variables. For instance, GDP alone had a weak explanatory effect on the distribution of the disease, but its explanatory effect was strengthened when coupled with the urbanization rate or local financial health expenditure. This indicates that among the 9 indicators, the urbanization rate should be the primary factor of concern. Additionally, after interaction, the factor combinations that achieved an explanatory degree with a q-value above 0.7 included residential population density (X_9_), expenditure on health care in local finances (X_2_), and urban basic medical insurance fund expenditure (X_4_). Although residential population density had a low individual impact on disease distribution, its explanatory effect significantly improved when coupled with economic indicators related to health care. This suggests that for HIV/AIDS incidence, residential population density should not be considered in isolation but should be simultaneously considered in conjunction with the synergistic effects of economic factors.

In terms of the type of enhanced interaction, apart from the nonlinear enhancement dominating the interactions of GDP (X_1_), per capita disposable income of residents (X_3_), and residential population density (X_9_), the interactions of the remaining 6 factors were predominantly bivariate enhancement. Furthermore, the detection results of factors X_1_, X_3_, and X_9_ were the weakest. Therefore, it is once again emphasized that GDP, per capita disposable income of residents, and residential population density exhibited relatively weak spatial distribution effects on HIV/AIDS incidence. However, it is important to note that these 3 factors are still relevant factors affecting HIV/AIDS incidence in China, albeit to a lesser extent compared to other factors.

## Discussion

This study conducted a spatiotemporal distribution analysis of HIV/AIDS incidence in 31 provinces of China from 2009 to 2019. Furthermore, it integrated the analysis with 9 economic and social indicators to explore the extent of the impact of socioeconomic factors on the spatiotemporal distribution of HIV/AIDS incidence in China. The research revealed that the occurrence of HIV/AIDS in China is significantly influenced by a combination of factors, including socioeconomic indicators, beyond the medical origins of the disease.

Before delving into various discussions, it is important to note that from the late 20th century to the early 21st century, China experienced localized outbreaks of HIV/AIDS in certain regions due to specific reasons. For instance, in Henan Province, from the late 1980s to the early 1990s, against the backdrop of official blood donation quotas, market demand from pharmaceutical companies, and the emergence of a blood-selling industry in rural areas, widespread transmission of HIV/AIDS occurred through blood products from rural blood stations due to technical and management flaws in blood collection. In August 2001, the Chinese Ministry of Health first publicized the situation of an “HIV/AIDS village” in Wenlou Village, Shangcai County, Henan Province, where around 43% of the approximately 3710 blood sellers were reported to be HIV positive [45]. In 2004, for the first time, Henan Province conducted a comprehensive HIV/AIDS census across the province. Out of the 280,000 individuals with a history of paid blood donation, 25,000 were found to be people living with HIV/AIDS [46]. Similarly, in Yunnan and Sichuan provinces, which serve as important land routes from southwestern China to Southeast Asia and South Asia, there was a relatively serious drug trafficking problem [47], leading to intravenous drug use becoming the main transmission route for HIV in these areas [48,49]. Existing research data have shown that before 2001, 74.8% of people living with HIV/AIDS identified in Sichuan Province were drug users, while 62.8% of those identified in Yunnan Province from 1989 to 2000 were drug users [50].

After the early 21st century, although the Chinese government increased its attention to the AIDS issue and gradually implemented corresponding prevention and control efforts (such as the “Four Frees and One Care” policy in 2003 and the promulgation of the HIV/AIDS Prevention and Control Regulation in 2006), the phenomenon of previous HIV/AIDS outbreaks gradually subsided. However, due to the large number of reported HIV/AIDS cases in China prior to these policies taking effect, coupled with a certain lag in policy implementation, HIV/AIDS prevention and control did not immediately curb the nationwide spread of HIV/AIDS. This also explains why the spatial distribution of HIV/AIDS incidence in China was random in 2009-2010 but gradually evolved from random distribution to clustered distribution from 2011 to 2019, as found in this study.

First, as a manageable chronic disease [51], the spatial distribution of HIV/AIDS is influenced by socioeconomic factors. For instance, the government’s investment in public health is one of the influencing factors. Since the end of 2003, China has implemented the “Four Frees and One Care” policy [10]. All people living with HIV/AIDS diagnosed at the CCDC can receive antiretroviral drugs for free. However, it was not until 2012, when important antiretroviral drugs like lamivudine (3TC) and efavirenz (EFV) gradually entered domestic production, that the government could progressively reduce the cost of obtaining drugs [52], thereby enhancing the practical implementation of the “Four Frees and One Care” policy in China. From the analysis of spatial distribution characteristics in this study, it is evident that although the incidence of HIV/AIDS in China from 2009 to 2019 exhibited continuous features of imbalance and concentration in spatial distribution, there was a significant turning point around 2012. This may be related to the continuous expansion of the coverage of the “Four Frees and One Care” policy. Subsequently, the results obtained from our application of the Geographical Detector to analyze the impact of medical factors once again indirectly corroborated this viewpoint. In this regard, it is necessary to acknowledge that some economically underdeveloped regions benefit more because the government can ensure the transfer and distribution of basic antiretroviral drugs (resulting in an increase in cold spots). However, due to differences in economic development and the proportion of public health expenditure among regions, there is a direct impact on the accessibility of new antiretroviral drugs [53]. This has a strong correlation with the spatiotemporal distribution differences in the incidence of HIV/AIDS. In simpler terms, in regions where the incidence of HIV/AIDS was relatively mild but economic development levels and public health expenditure were low, the comprehensive implementation of the “Four Frees and One Care” policy in 2012 ensured the right of people living with HIV/AIDS to receive free antiretroviral drugs. Although subsequent public health challenges in HIV/AIDS prevention and control still exist in these areas, considering the initially low disease burden, the overall incidence is expected to further decrease. In regions where the HIV/AIDS epidemic was already severe, after 2012, they faced more acute local financial difficulties. The lack of momentum in public health expenditure directly results in a large number of people living with HIV/AIDS who are unable to access quality medical resources and new antiretroviral drugs. Consequently, the incidence in these areas has not reached the desired level of control. This finding suggests that, in future reforms of HIV/AIDS prevention and control policies, the Chinese government should pay attention to balancing the relationship between policy and local economic conditions.

Furthermore, parallel to the “Four Frees and One Care” policy, there is an ongoing reform of China’s medical insurance system. In March 2009, China issued the “Opinions on Deepening the Reform of the Medical and Health System” [54], marking a period of improvement in China’s medical security system. This is one of the reasons why this study chose 2009 as the starting point for data observation. In 2012, several government departments in China jointly issued a document requiring the comprehensive implementation of total control over medical insurance payments [55]. This led to a significant increase in the pressure on local public health expenditure at the regional level. This situation is intertwined with the regional disparities in providing free antiretroviral drugs under the “Four Frees and One Care” policy. However, it is crucial to note that there is no absolute proportional relationship between local GDP and the intensity of investment in public health [56]. In other words, a higher GDP in a certain region does not necessarily mean a synchronous increase in investment in public health. This is also clearly reflected in the previous data of this study. However, in 2016, the Chinese Ministry of Human Resources and Social Security, along with the Ministry of Finance, officially initiated the direct settlement of inpatient medical expenses across regions [57], facilitating the access of the public to medical care across different areas. This development is consistent with the earlier findings of our analysis, indicating that from 2016 to 2019, the spatial and temporal distributions of HIV/AIDS incidence in China stabilized and the impact of factors, such as population density and per capita disposable income, on the HIV/AIDS incidence rate decreased. However, despite the potential enhancement of shared health care resources across regions, the actual economic burden borne by individuals still exhibits significant regional economic disparities. For instance, the contribution base for medical insurance, which is closely tied to local fiscal investment in public health, continues to vary markedly across regions [58]. This may explain why, after 2016, the disparity in HIV/AIDS incidence rates among different regions in China did not widen or narrow. This finding suggests a direction for the Chinese government to prioritize and strengthen its focus on certain indicators in the ongoing efforts to combat the HIV/AIDS epidemic.

Looking further, how do these indicators specifically and directly relate to the incidence of HIV/AIDS among individuals, as mentioned previously? First, in terms of economic indicators, the current “Four Frees and One Care” policy provides a combination of free antiretroviral drugs that are categorized into first-line (tenofovir disoproxil fumarate [TDF]/azidothymidine [AZT]/abacavir [ABC]+3TC+nevirapine [NVP]/EFV/rilpivirine [RPV]) and second-line (TDF+3TC+dolutegravir [DTG]/lopinavir combined with ritonavir [LPV/r]; AZT+TDF+3TC+DTG/LPV/r) treatments [52]. Typically, first-line drugs are centrally purchased by the CCDC and systematically distributed to various regions. As for second-line drugs, their availability primarily relies on the level of financial investment by local governments in public health care. This directly results in situations where drugs like LPV/r, previously categorized as a second-line treatment, are not widely accessible in all regions. In comparison with numerous antiretroviral drugs, DTG, which is considered to have fewer side effects and lower susceptibility to resistance, is more representative [59]. Currently, access to DTG for individuals in different regions can be categorized in 3 ways. First, it is directly obtained for free through the “Four Frees and One Care” policy in certain regions (such as cold spot areas like Beijing). Second, in areas where DTG is included in medical insurance, infected individuals with insurance coverage can purchase DTG and be reimbursed through insurance (such as Zhejiang) [60]. Third, in regions where DTG is not covered by medical insurance, infected individuals have to self-fund the purchase, and in some cases, they have to resort to ordering it through mail from other regions (such as Sichuan and other hot spot areas) [61]. It is evident that economic indicators in different regions influence whether people living with HIV/AIDS can access antiretroviral drugs for free or acquire updated and more effective medications at lower costs, thereby affecting the incidence of HIV/AIDS among individuals.

Certainly, another crucial factor in preventing the onset of the disease is the timely detection of treatment failure (such as drug resistance) in people living with HIV/AIDS. This is closely related to the social indicators mentioned in this study. In our research, we observed that regions with higher levels of health care resources are more effective at suppressing the incidence of HIV/AIDS. In different regions, there are significant variations in whether people living with HIV/AIDS need routine examinations and whether the costs of such checks, which are conducted each time they receive free antiretroviral drugs under the “Four Frees and One Care” policy (as stipulated by the policy, approximately once every 3 months), need to be self-funded by the individuals. In some regions with more developed public health services, there are even provisions to offer 1 to 2 free drug resistance testing services annually for individuals [52]. In other words, in regions with more abundant public health care resources, individuals are more likely to timely detect their drug-resistance situations, enabling targeted changes in antiretroviral drug combinations and thus preventing the onset of the disease. In addition, it is worth noting that China has implemented the “designated hospital” system for HIV/AIDS medical care, as outlined in the 2013 “Notice on Submitting the List of Designated Hospitals for AIDS by the Medical Administration Bureau of the Ministry of Health” [62]. While this system aims to reduce urban-rural disparities in implementing the “Four Frees and One Care” policy, its effectiveness has been a subject of mixed opinions and has, to some extent, led to instances of nondesignated hospitals refusing to provide medical services. A region that is more densely populated, economically developed, and well-endowed with public medical resources usually has more numerous and specialized designated hospitals, with a high degree of professionalism and comprehensive capabilities. For instance, hospitals like You'an Hospital and Ditan Hospital in Beijing (a cold spot region) have become the “most renowned” designated hospitals for people living with HIV/AIDS in the country. In contrast, designated hospitals in regions with relatively scarce public medical resources mainly rely on infectious disease specialist hospitals as they lack comprehensive departmental setups. This directly results in medical challenges for individuals living with HIV/AIDS in those areas. In fact, before the onset of the disease, the medical needs of people living with HIV/AIDS are not limited to the field of infectious diseases, similar to the general population [63]. However, given the vulnerability of the immune system [64], if people living with HIV/AIDS do not receive timely and effective medical services, they are more susceptible to overall health deterioration due to other diseases. This also demonstrates the significant impact of medical accessibility on the onset of the disease. In the future, the Chinese government should continue to optimize medical resource allocation for this specific group of people living with HIV/AIDS to address the public health challenges posed by the HIV/AIDS epidemic.

Finally, this study not only revealed the spatiotemporal distribution of HIV/AIDS incidence in China as a result of the comprehensive impact of multiple influencing factors through the interactive detection of geographic detectors but also discovered that the dual-factor coupling of 9 socioeconomic indicators can have different effects on the spatiotemporal distribution of HIV/AIDS incidence in China. This finding can provide insights for the Chinese government to focus on the combined optimization of socioeconomic indicators when formulating HIV/AIDS prevention and control policies. Specifically, when 2 indicators show a significantly improved coupling effect, the government can enact policies to promote the development of the optimal indicator combination to further reduce the incidence of HIV/AIDS. For example, in the case where the coupling effect of the urbanization rate with other indicators is most noticeable, the government can actively formulate policies to leverage the synergistic effects between this indicator and others while continuing to promote urbanization.

This study has certain limitations. First, due to the lack of authoritative data statistics on the incidence of HIV/AIDS in Hong Kong, Macao, and Taiwan by official institutions, this study used relevant data from 31 other provinces in China. Second, the socioeconomic indicators used in this study are macro and common statistical indicators, but the causes of HIV/AIDS are complex and diverse, and these indicators may not necessarily cover all influencing factors. To overcome these limitations, future efforts should focus on collecting more detailed data and interpreting them in the context of emerging research. However, as one of the few studies addressing this topic, our research covers the majority of China’s regions, and the analyzed content and recommendations have a relatively high degree of universality.

This study conducted a spatiotemporal distribution analysis of HIV/AIDS incidence in China from 2009 to 2019. The findings revealed that, starting from 2012, there was an enhancement in the clustering pattern of HIV/AIDS incidence in China, with cold and hot spots becoming more pronounced. From 2016 to 2019, the clustering of cold and hot spots began to stabilize. Additionally, we investigated the impact of HIV/AIDS incidence from 2009 to 2019 in relation to 9 common socioeconomic indicators. The study findings suggest that optimizing the combination of different socioeconomic indicators can further suppress the incidence of HIV/AIDS. These findings provide insights for the government to adjust relevant policies based on the specific impact of different indicators on HIV/AIDS incidence in the future.

## Abbreviations

3TC: lamivudine
ART: antiretroviral therapy
AZT: azidothymidine
CCDC: Chinese Center for Disease Control and Prevention
CNBS: China National Bureau of Statistics
CPHSD: Chinese Public Health Science Data Center
DTG: dolutegravir
EFV: efavirenz
GDP: gross domestic product
IRB: Institutional Review Board
LPV/r: lopinavir combined with ritonavir
NVP: nevirapine
RPV: rilpivirine
TDF: tenofovir disoproxil fumarate
UNAIDS: Joint United Nations Program on HIV/AIDS

## Data Availability

The data sets analyzed during this study are available in the Chinese Public Health Science Data Center repository [65].
